# Rapid identification of feline sporotrichosis by ATR-FTIR spectroscopy coupled with machine learning

**DOI:** 10.1007/s00216-026-06671-3

**Published:** 2026-07-24

**Authors:** Eric Luiz Domingos, Ahmad Kassem El Zein, Monica Surek, Haroldo Greca Junior, Izabella Castilhos Ribeiro dos Santos-Weiss, Luana Mota Ferreira, Roberto Pontarolo

**Affiliations:** 1 Postgraduate Program in Pharmaceutical Sciences, Universidade Federal Do Paraná, 623, Prefeito Lothario Meissner Avenue, Jardim Botânico, Curitiba, Paraná 80210-170 Brazil; 2 Department of Pharmacy, Universidade Federal Do Paraná, 623, Prefeito Lothario Meissner Avenue, Jardim Botânico, Curitiba, Paraná 80210-170 Brazil; 3 Embrapa Semiárido, BR-428 Highway, Km 152, Rural Area, Petrolina, Pernambuco Brazil; 4 Zoonosis Surveillance Unit, Secretaria Municipal de Saúde de São José Dos Pinhais, 237, Quirino Zagonel Street, São José Dos Pinhais, Braga, Paraná Brazil; 5 Department of Clinical Analysis, Universidade Federal Do Paraná, 623, Prefeito Lothario Meissner Avenue, Jardim Botânico, Curitiba, Paraná Brazil

**Keywords:** Feline sporotrichosis, ATR-FTIR spectroscopy, Diagnostic model, Machine learning, One Health

## Abstract

Feline sporotrichosis is a zoonotic subcutaneous mycosis of major relevance in endemic regions of Latin America, particularly in Brazil. Current diagnostic methods are limited by turnaround time, specificity, and cost. In this context, spectroscopy combined with machine learning has emerged as a promising alternative. This study aimed to develop and validate predictive models for the rapid and minimally invasive diagnosis of feline sporotrichosis using ATR-FTIR spectroscopy applied to plasma samples. Seventy-five cats were included and classified by fungal culture as the reference standard. Plasma samples were analyzed by ATR-FTIR (4000–400 cm⁻^1^), with 20 spectra acquired per animal. Data were evaluated using six machine learning algorithms under three validation frameworks: spectral-level internal testing, Leave-One-Patient-Out (LOPO) cross-validation, and independent external validation (*n* = 15 animals). On the internal test set, Random Forest, KNN, and LightGBM showed similarly strong discriminative ability (AUC ≥ 0.998), with no significant differences among them. The LOPO cross-validation confirmed biological generalization, with SVM achieving the highest AUC (0.896). External validation confirmed consistent performance, with Random Forest and LightGBM achieving an AUC of 1.000 and excellent probabilistic calibration (low Brier scores). Spectral variable importance and SHAP analyses revealed that discrimination was primarily driven by regions associated with carbohydrates (1040–1050 cm⁻^1^) and lipids (1746–1751 cm⁻^1^), reflecting the host’s systemic metabolic and immunological response to the infection. These findings suggest that ATR-FTIR combined with machine learning provides consistent and generalizable diagnostic performance, representing a rapid and minimally invasive approach within the One Health framework.

**Graphical abstract:**

**Figure:**
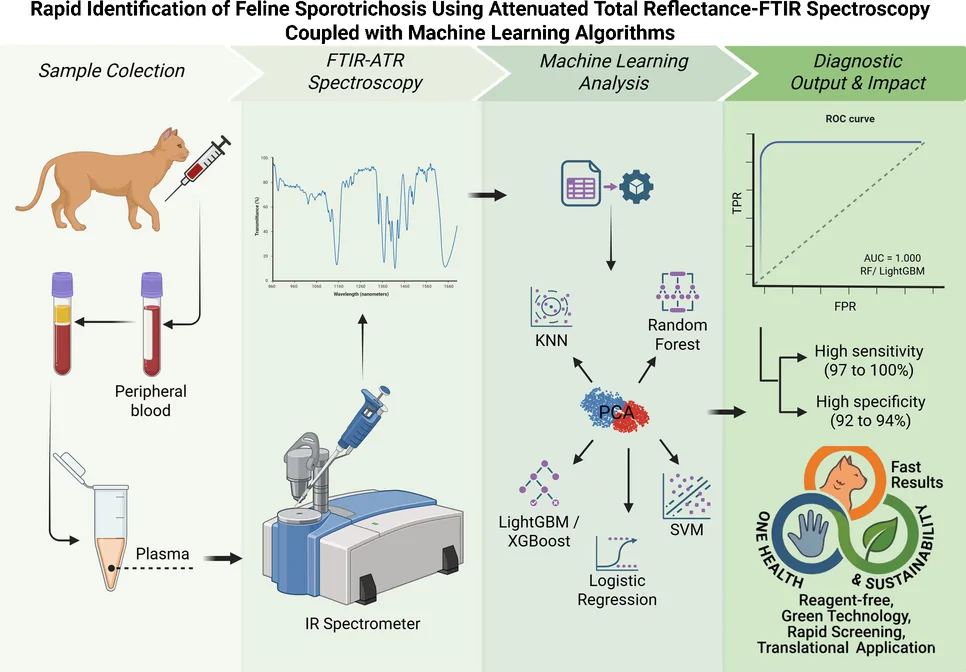

**Supplementary Information:**

The online version contains supplementary material available at https://doi.org/10.1007/s00216-026-06671-3.

## Introduction

Feline sporotrichosis is a neglected subcutaneous mycosis caused by fungi of the genus *Sporothrix*, with *Sporothrix brasiliensis* being the most prevalent and virulent species, particularly in Latin America [1, 2]. Domestic or free-roaming cats are highly susceptible to *S. brasiliensis* and *S. schenckii*, playing a crucial role as vectors and reservoirs of the disease, with zoonotic transmission to humans [3–5].

Clinically, the disease may range from localized cutaneous lesions to fatal systemic forms. Common manifestations include ulcerative lesions that frequently affect the head, particularly the nasal and auricular areas, limbs, and the base of the tail. Respiratory involvement (sneezing, dyspnea, and nasal discharge) and mucosal lesions are prevalent findings and are associated with an increased risk of treatment failure and death. Infected cats generally exhibit an elevated fungal burden in their lesions, which facilitates transmission [2, 6, 7].

Transmission occurs primarily through horizontal spread, via scratches and bites from infected cats, especially in endemic areas. Intact male cats with outdoor access play a significant role in the dissemination of the infection [6, 8–10]. Feline sporotrichosis represents a major concern in endemic regions of South America, particularly in Argentina, Brazil, Colombia, Mexico, Panama, Paraguay, and Peru. Brazil reports the highest number of cases worldwide, with epidemics occurring since 1998 affecting both felines and humans [10–17].

Feline sporotrichosis presents a complex and likely underestimated epidemiological scenario due to the lack of standardized surveillance and underreporting, which limits robust estimates of prevalence and incidence [18, 19]. Additionally, asymptomatic or subclinical animals may act as sources of infection, contributing to the maintenance of transmission within the population [20]. The geographic expansion of the disease, driven mainly by *S. brasiliensis*, shows its environmental adaptability and reinforces its emerging zoonotic character in different regions [21, 22]. The most common epidemiological profile involves adult, mixed-breed, intact male cats. The increase in cat cases generally precedes the rise in human cases, underscoring the importance of early diagnosis and control measures for public health [5, 7, 10].

The reference standard for the diagnosis of feline sporotrichosis is fungal isolation through culture; however, this test may take up to 30 days to yield results. To expedite diagnosis, cytopathological examination may be employed and has demonstrated sensitivity ranging from 78% to 89.4%. Despite this high sensitivity, the technique presents limited specificity, and its diagnostic accuracy is strongly dependent on the analyst’s expertise [23]. Other approaches include histopathology with Grocott staining (GSS) and immunohistochemistry (IHC). Cell block cytology (CBLC) has also been shown to be an efficient and rapid tool, as have serological tests (ELISA), which detect specific antibodies. Molecular techniques, such as Polymerase Chain Reaction (PCR), offer the highest accuracy, with 90% sensitivity and 94% specificity. However, the significant cost, technical complexity, and the possibility of false-negative results due to low fungal burden in the sample represent important challenges [23]. Therefore, there is a need to develop more accessible and rapid diagnostic alternatives to improve early detection, facilitate treatment, and control disease dissemination.

Beyond diagnostic performance, the development of novel diagnostic strategies should also consider sustainability and environmental impact. Conventional laboratory methods often require reagents, amplification kits, staining chemicals, and generate biological and chemical waste [24]. Infrared spectroscopy aligns with principles of green analytical chemistry by emphasizing the minimization of reagents, energy consumption and hazardous waste, and has been highlighted as a direct, low-preparation technique suitable for clinical biofluids [25–27]. Attenuated total reflectance in Fourier-transform infrared spectroscopy (ATR-FTIR) in particular requires minimal sample preparation, no chemical reagents for analysis, and low consumable use, which supports lower analytical footprints compared with reagent-intensive molecular assays [25]. For zoonotic diseases, particularly those affecting companion animals with direct public health implications, the adoption of environmentally sustainable and ethically responsible diagnostic technologies reinforces the One Health perspective, integrating animal welfare, human health, and environmental preservation [28].

ATR-FTIR emerges as a promising technique, as it generates a spectral “fingerprint” of biomolecules in samples such as blood plasma [29, 30]. Recently, spectroscopic techniques combined with machine learning algorithms have been explored for the diagnosis of conditions such as Hansen’s disease [31], arboviral diseases [32], cancer diagnosis [33, 34], trichuriasis [35], and COVID-19 [36–38]. These approaches have achieved promising diagnostic performance metrics. Current available methods for feline sporotrichosis, however, remain dependent on highly complex laboratory infrastructure or prolonged turnaround times.

Despite advances in spectroscopy for human diseases, the application of ATR-FTIR for the diagnosis of zoonotic mycoses in veterinary medicine remains incipient. Previous studies have primarily focused on taxonomic identification from pure cultures, which does not address the diagnostic time bottleneck. Therefore, the aim of this study is to develop and validate a predictive model for a rapid and non-invasive diagnosis of feline sporotrichosis using ATR-FTIR spectroscopy in plasma samples, in combination with machine learning algorithms.

## Materials and methods

### Materials

Blood collection microtubes containing disodium EDTA (Labor, Brazil), 1 mL syringes (BD, Brazil), 20 × 0.55 mm hypodermic needles (24G) (BD, Brazil), and 24G scalp vein sets (BD, Brazil), 2 mL centrifuge microtubes (Eppendorf, Germany), Mycosel™ agar (BD, France), sterile dry swabs without transport medium (Cralplast, Brazil), and lactophenol cotton blue (Exodo, Brazil) were purchased from local suppliers.

### Study design, ethical considerations, and animal selection

This study involves the development and validation of a diagnostic model for feline sporotrichosis based on spectral data obtained through ATR-FTIR spectroscopy and analyzed using machine learning models. The authors confirm that the journal’s ethical policies, as described in the guidelines for authors, were fully respected and that appropriate ethical approval was obtained prior to the initiation of the study.

The project was approved by the Animal Ethics Committee for Research of the Agrarian Sciences Sector of the Federal University of Paraná (UFPR), under protocol no. 069/2023. All study procedures were conducted in strict accordance with the International Guiding Principles for Biomedical Research Involving Animals, established by the Council for International Organizations of Medical Sciences [39].

Sample collection was performed at veterinary clinics located in the municipalities of Curitiba and São José dos Pinhais, Brazil, by an experienced and duly licensed veterinarian. All legal guardians of the animals received detailed information regarding the procedures and agreed to participate in the study by signing the Informed Consent Form (ICF).

**Fig. 1:**
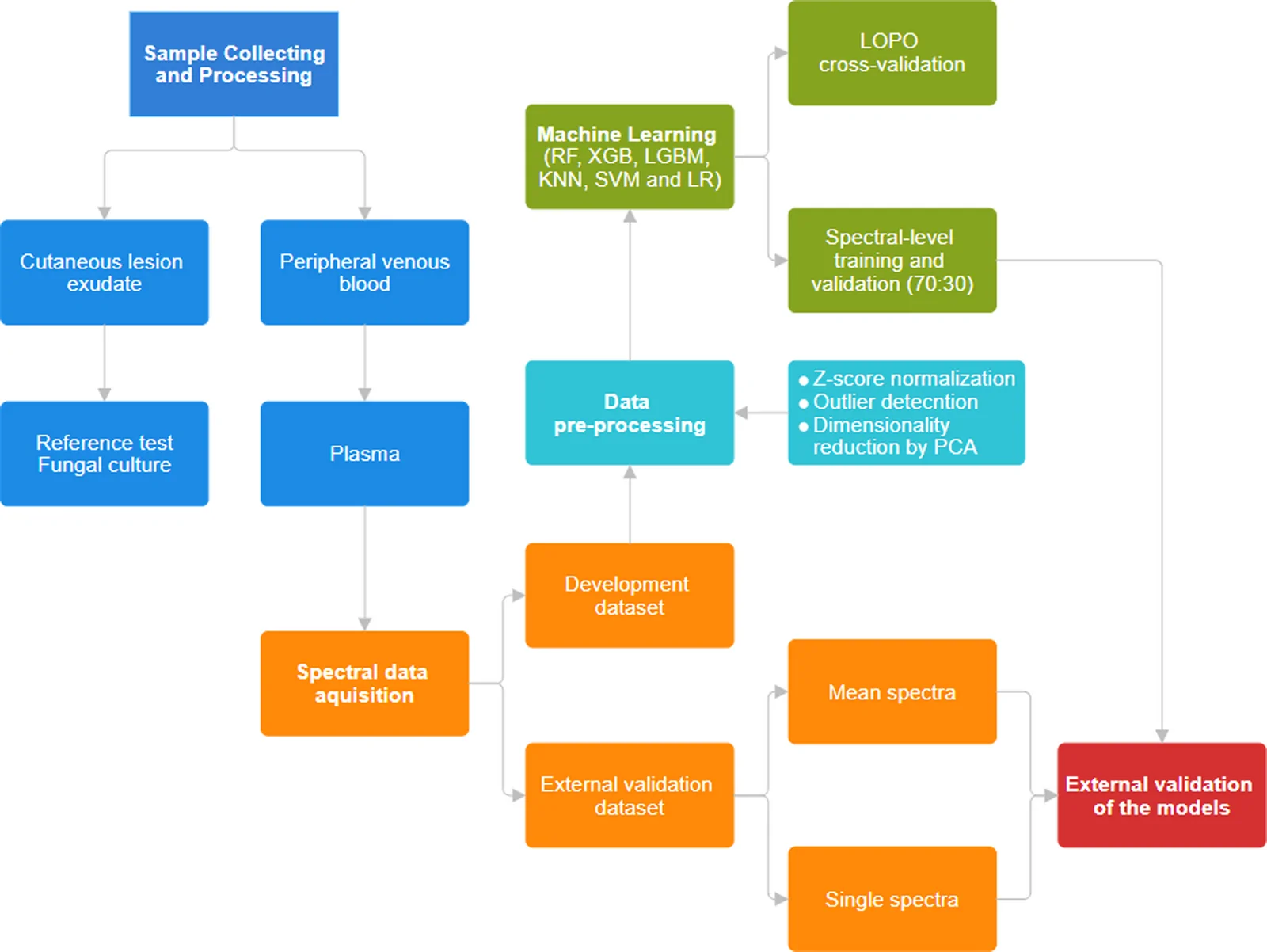
Flowchart of the experimental design and analytical pipeline. KNN, K-Nearest Neighbors; LGBM, Light Gradient Boosting Machine; LOPO, Leave-One-Patient-Out; LR, Logistic Regression; PCA, Principal Components Analysis; RF, Random Forest; SVM, Support Vector Machines; XGB, Extreme Gradient Boosting

Cats were included without restriction regarding age, sex, or management condition (domestic or feral). All animals underwent prior clinical and epidemiological evaluation and were classified as either infection-free (negative controls) or clinically suspected of sporotrichosis. The overall methodological workflow, from animal selection and sample processing to spectral acquisition and machine learning validation, is summarized in Fig. 1.

### Sample collection and processing

For each animal included in the study, peripheral venous blood and cutaneous lesion exudate samples were collected. Blood collection was performed using a syringe fitted with a 20 × 0.55 mm hypodermic needle (24G) or a 24G scalp vein set. A volume of 1 mL of blood was collected and immediately transferred into tubes containing disodium EDTA as an anticoagulant.

Blood samples were centrifuged at 1,369 × g for 10 min at 20 °C to separate plasma from the cellular components. The obtained plasma was transferred to centrifuge tubes previously labeled with the animal’s identification code and stored frozen at − 40 °C until spectral analyses were performed.

Exudate from cutaneous lesions was collected using a sterile dry swab without transport medium and maintained at room temperature until inoculation onto appropriate culture medium.

### Fungal culture

Material collected using a swab was inoculated onto Mycosel agar and incubated at 25 °C for up to 30 days. Samples that showed no fungal growth or exhibited colony growth not compatible with the genus *Sporothrix* were considered negative.

Samples that exhibited colony growth with macroscopic morphology suggestive of *Sporothrix* spp. were subjected to microscopic evaluation using lactophenol cotton blue staining for morphological confirmation. Characteristic colonies that grew in association with other unidentified colonies were subcultured for isolation and subsequent confirmation. Fungal culture was considered the diagnostic reference standard for classifying samples as positive or negative for sporotrichosis.

### Spectral data acquisition

Mid-infrared spectra were acquired using a Bruker Alpha P FTIR spectrometer (Bruker OPTIK GmbH, Ettlingen, Germany) equipped with an attenuated total reflectance (ATR) accessory and operated using OPUS software (version 6.5). Aliquots of 25 µL of plasma were deposited onto the diamond crystal and analyzed in the liquid state. Spectra were collected over the 4000–400 cm⁻^1^ spectral range using 64 accumulated scans at a spectral resolution of 4 cm⁻^1^, conditions selected to provide an adequate signal-to-noise ratio while preserving spectral resolution. Before analyzing each sample, a new background spectrum was recorded and automatically subtracted to minimize interference from atmospheric moisture and carbon dioxide. The entire area of the deposited sample was analyzed to improve spectral representativeness and reduce the influence of local compositional heterogeneity. To assess analytical repeatability and capture potential variability associated with sample deposition, crystal contact, and instrumental acquisition, each plasma sample was measured in 20 independent replicates. This strategy generated a comprehensive spectral dataset, allowing for the evaluation of the reproducibility of disease-associated spectral signatures in repeated measurements. Spectral acquisitions were performed according to sample availability and laboratory workflow. Instrument operating conditions, acquisition parameters, and spectral collection procedures were kept constant throughout the study to minimize analytical variability between measurements.

### Data processing

Prior to model training, the raw data were standardized using Z-score normalization to ensure that variables with different magnitudes did not introduce bias into the algorithms. To maintain scientific integrity and prevent data leakage, variable standardization and dimensionality reduction procedures were fitted exclusively using the statistical parameters from the training set and subsequently applied to the test and validation datasets.

### Machine learning model development

Model construction and feature handling were implemented in Python (version 3.12.13, Python Software Foundation, https://www.python.org) using the following libraries and versions: *scikit-learn* (1.6.1), *pandas* (2.2.2), *NumPy* (2.0.2), SciPy (1.16.3), *Matplotlib* (3.10.0), *XGBoost* (3.2.0), *LightGBM* (4.6.0), *UMAP-learn* (0.5.12), and *Joblib* (1.5.3). Separate datasets were used for development and external validation. Multiple spectra were acquired from each animal. Therefore, two complementary validation strategies were adopted. The first strategy consisted of spectral-level training and validation, in which individual spectra were treated as analytical observations. The dataset was divided into training and test subsets using stratified sampling to preserve class proportions in both partitions. Hyperparameter optimization was performed using nested cross-validation within the training set, and model performance was evaluated on the reserved test subset. This approach was designed to assess the ability of machine learning algorithms to discriminate spectral patterns associated with feline sporotrichosis and to evaluate the reproducibility of disease-related spectral signatures across repeated measurements.

The second strategy consisted of Leave-One-Patient-Out (LOPO) cross-validation, which was employed to evaluate model generalization at the biological level while accounting for the dependence among spectra acquired from the same animal. In each iteration, all spectra obtained from a single animal were assigned to the test set, whereas spectra from all remaining animals were used for model training. This procedure was repeated until every animal in the development dataset had been used once as the test set, and predictions from all iterations were aggregated to calculate overall performance metrics. By ensuring complete separation between animals used for training and evaluation, this strategy provided a more conservative and clinically relevant estimate of model generalization to previously unseen individuals.

The binary outcome was defined as the presence or absence of sporotrichosis confirmed by fungal culture. Predictors consisted of spectral absorbance values in the mid-infrared region. Prior to model training, spectra were standardized using Z-score normalization, and dimensionality reduction was performed using PCA. Outlier detection, standardization, PCA fitting, and all subsequent preprocessing procedures were performed exclusively on the training data and then applied to the corresponding test partitions to ensure strict separation between training and testing data.

Several supervised machine learning algorithms were evaluated, including Random Forest (RF), Extreme Gradient Boosting (XGBoost), Light Gradient Boosting Machine (LightGBM), K-Nearest Neighbors (KNN), Support Vector Machines (SVM), and Logistic Regression (LR). Hyperparameter optimization was performed through nested cross-validation with grid search in the inner loop. Performance estimates were obtained from both spectral-level validation and LOPO validation, allowing assessment of analytical discrimination and animal-level generalization, respectively.

External validation was performed using an independent dataset not involved in any stage of model development. To assess performance under different clinically relevant scenarios, two complementary approaches were adopted. First, all spectra acquired from each animal were averaged to generate a representative spectrum. Second, a single spectrum was randomly selected from each animal to simulate a clinical workflow in which only one spectral acquisition is available for decision-making. The previously trained preprocessing and classification pipeline was applied without re-optimization.

### Evaluation of machine learning model performance

Model performance was evaluated with the objective of assessing discriminative ability, calibration, and classification robustness using standard classification metrics. These included accuracy, sensitivity (recall), specificity, precision, F1 score, Cohen’s Kappa coefficient, and the Matthews Correlation Coefficient (MCC). Sensitivity, precision, and accuracy were calculated as follows:

$$Sensitivity=\frac{TP}{TP+FN}$$

$$Precision=\frac{TP}{TP+FP}$$

$$Accuracy=\frac{TP+TN}{TP+TN+FP+FN}$$

where

TP: True positives

TN: True negatives

FP: False positives

FN: False negatives

The F1 score, Cohen’s Kappa, and MCC were included to provide a balanced evaluation of model performance, particularly considering the potential influence of class distribution. Among these, MCC was prioritized as a robust metric that incorporates all elements of the confusion matrix and provides a reliable summary of classification performance. Discriminative performance was quantified using the area under the receiver operating characteristic curve (AUC-ROC). Receiver operating characteristic (ROC) curves were constructed to evaluate the trade-off between sensitivity (true positive rate) and 1 − specificity (false positive rate). Model performance was further explored through confusion matrices and class-specific error patterns.

Calibration performance was assessed using the Brier Score, as well as calibration intercept and slope, to evaluate the agreement between predicted probabilities and observed outcomes. To quantify uncertainty, 95% confidence intervals (95% CI) for primary performance metrics, including AUC-ROC, sensitivity, specificity, and F1 score, were estimated using bootstrap resampling with 2000 iterations.

Statistical comparison of discriminative performance between models was conducted using DeLong’s test for correlated ROC curves. Initially, a global (omnibus) test was applied to assess whether significant differences existed among all evaluated models. Subsequently, pairwise DeLong tests were performed to compare models individually. A significance level of α = 0.05 was adopted. Given the multiple pairwise comparisons, results were interpreted with caution regarding the potential for type I error inflation. All statistical comparisons using DeLong’s test were performed at the spectral level and should be interpreted considering the potential intra-individual dependence arising from multiple spectra per animal.

To ensure reproducibility, all analyses were conducted using a fixed random seed (42). The sample size was determined by convenience based on clinical availability, and a post hoc power assessment was performed to evaluate the ability to detect differences in discriminative performance given the observed effect sizes.

## Results

### Sample characterization

A total of 75 cats were included, with a mean age of 2.94 years (SD = 2.12), of which 39.2% (*n* = 29) were male. Most animals were not neutered (70.3%; *n* = 52). Based on fungal culture, considered the diagnostic reference standard, 48.6% (*n* = 36) were classified as positive for sporotrichosis (Table 1). These animals generated 1500 ATR-FTIR spectra through 20 technical replicates per sample. Sixty animals (1200 spectra) comprised the development dataset, whereas 15 animals (300 spectra) were reserved exclusively for external validation. For spectral-level validation, the development dataset was divided into training (70%) and internal testing (30%) subsets while preserving class proportions. No animal included in the external validation dataset contributed to model development, hyperparameter optimization, or internal validation.

**Table 1:** Description of epidemiological and clinical variables of cats

	Negative control	Positive	Total
n (%)	39 (52)	36 (48)	75
Average age in years ± SD	2.88 ± 1.74	2.96 ± 2.01	2.94 ± 2.12
Males (%)	10 (25.6)	19 (52.8)	29
Not neutered (%)	39 (100)	14 (38.9)	52
Contact with an animal with sporotrichosis (%)	0 (0)	15 (41.7)	15
Presence of any lesion compatible with sporotrichosis (%)	0 (0)	28 (77.8)	28

*%*, percentage; *n*, categorical variables as absolute number; *SD*, standard deviation

### Spectral and principal component loading analysis

**Fig. 2:**
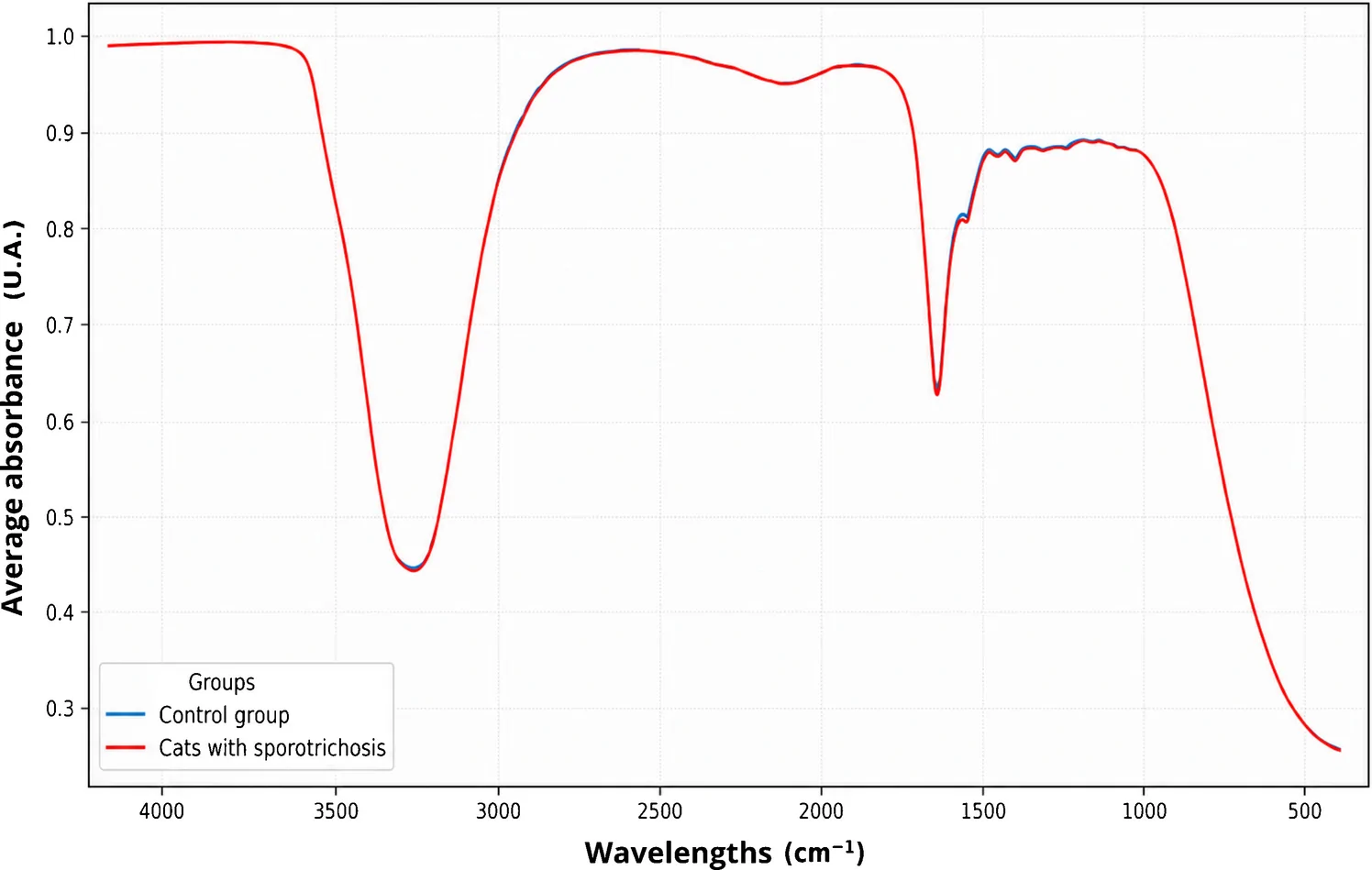
Mean ATR-FTIR spectra of plasma samples from control cats and cats with sporotrichosis

As detailed in Fig. 2, the mean ATR-FTIR spectra were characterized by broad and intense absorption bands distributed throughout the mid-infrared region. A prominent broad band was observed between 3600 and 3000 cm⁻^1^, accompanied by an intense absorption centered near 1650 cm⁻^1^ and several moderate bands within the fingerprint region between 1500 and 900 cm⁻^1^.

The broad absorption observed in the 3600–3000 cm⁻^1^ region is predominantly attributed to O–H stretching vibrations of water present in the sample, which are strongly broadened by the extensive intermolecular hydrogen-bonding network characteristic of aqueous systems. However, additional contributions from N–H and O–H vibrations associated with other matrix constituents cannot be completely excluded [40, 41].

A well-defined spectral feature was identified in the 1746–1751 cm⁻^1^ region, corresponding primarily to C = O stretching vibrations of carbonyl-containing structures. Another important absorption was observed between 1650 and 1600 cm⁻^1^, corresponding to conjugated C=O and C=C vibrations, as well as deformation modes involving nitrogen-containing functional groups [42].

Additional spectral information was observed between 1560 and 1450 cm⁻^1^, where several bands of moderate intensity are present. These absorptions are generally attributed to C–H and N–H deformation modes, as well as mixed vibrations from a variety of organic functional groups, providing structural information complementary to the intense band centered near 1650 cm⁻^1^ [43].

Evaluating the first 3 principal components, PC1 explained 46.78% of the total spectral variance and was predominantly associated with bands located between 3003 and 3043 cm⁻^1^, which may correspond to the C–H stretching vibrations commonly attributed to lipid-related molecular structures. PC2 explained 17.69% of the variance and was mainly influenced by low-frequency variables, between 477 and 592 cm⁻^1^. PC3 accounted for 11.01% of the variance and was dominated by bands located at 1620–1630 cm⁻^1^, 1554–1564 cm⁻^1^, and 1456–1458 cm⁻^1^, corresponding to the Amide I, Amide II, and CH_2_ strain regions, respectively [43]. Together, the first three principal components explained 75.48% of the total spectral variance (see Supplementary Material - Figure A1 and Figure A2).

PCA score plots demonstrated partial clustering between positive and negative animals, although substantial overlap remained, indicating that disease-related biochemical variation is subtle and distributed across multiple spectral variables. This observation supports the subsequent use of supervised learning algorithms for extracting diagnostically relevant information from the spectral dataset.

### Model development and internal performance

A total of 1200 spectra from the development dataset were processed through the machine learning pipeline. Following Z-score standardization, outlier detection, and dimensionality reduction using PCA, 18 anomalous spectra were identified and removed from the training set prior to model construction (see Supplementary Material - Figure A2). Optimization of the PCA model identified 19 principal components as the optimal configuration, accounting for 98.33% of the total variance.

**Fig. 3:**
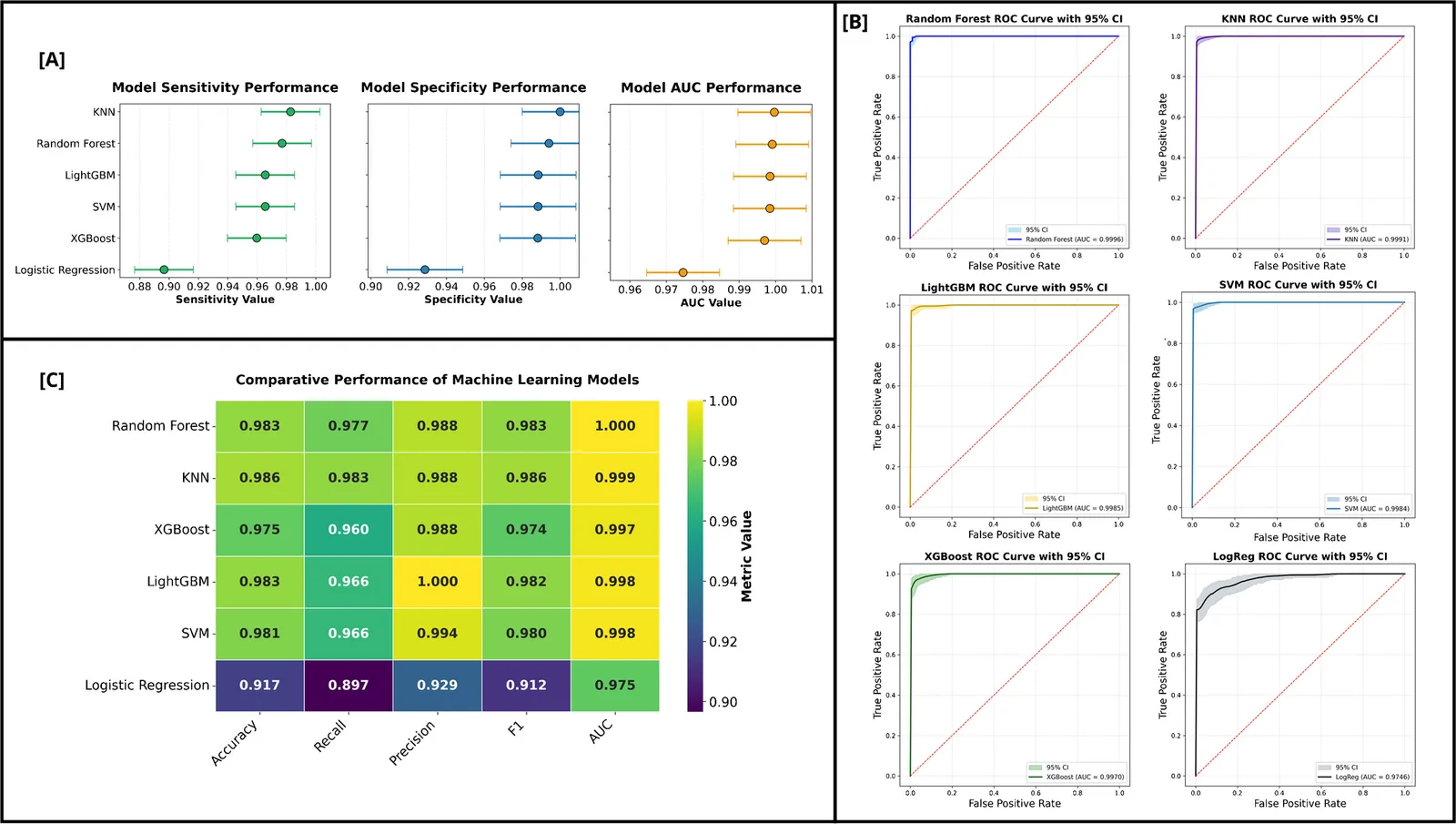
Internal test set performance of machine learning models based on plasma ATR-FTIR data. Comparative performance analysis of machine learning models in the internal independent test set, including **A** Sensitivity, specificity, and AUC performance with 95% CI, **B** ROC curves, and **C** overall classification metrics. AUC, area under the curve; FPR, 1 − specificity; MCC, Matthews Correlation Coefficient; TPR, sensitivity

The supervised machine learning algorithms demonstrated satisfactory and comparable performance on the independent internal test set (Table 2; Fig. 3). Specifically, KNN, Random Forest, and LightGBM demonstrated excellent and statistically equivalent classification capabilities. As detailed in Table 2, these three models showed overlapping results across all evaluated metrics, achieving good discrimination (AUC) and satisfactory classification balance (MCC).

**Table 2:** Performance of the models on the independent test set

	Accuracy	AUC	Recall	Precision	F1-score	Kappa	MCC
Random forest	0.983	1.000	0.977	0.988	0.983	0.967	0.967
KNN	0.986	0.999	0.983	0.988	0.986	0.972	0.972
LightGBM	0.983	0.998	0.966	1.000	0.982	0.966	0.967
SVM	0.981	0.998	0.966	0.994	0.980	0.961	0.961
XGBoost	0.975	0.997	0.966	0.982	0.974	0.945	0.950
Logistic regression	0.917	0.975	0.897	0.929	0.912	0.833	0.833

*AUC*, area under the curve; *MCC*, Matthews Correlation Coefficient

The global comparison of ROC curves using DeLong’s test indicated no statistically significant difference among the evaluated models (χ^2^ = 0.2016, df = 5, p = 0.9991), supporting equivalent discriminative performance. Pairwise comparisons confirmed that differences among the top-performing models (KNN, Random Forest, and LightGBM) were not statistically significant (p > 0.05). In contrast, Logistic Regression showed significantly lower discriminative performance compared to all other models (p < 0.001), while XGBoost showed a small yet statistically significant difference compared to Random Forest (p = 0.04099), although this difference is numerically minimal.

The analysis of generalization performance revealed minimal discrepancies between training and internal test AUC values across all models (Table 3), indicating a low likelihood of overfitting. However, calibration analysis highlighted distinct probabilistic behaviors among the algorithms. As detailed in Table 3, KNN and LightGBM achieved the lowest Brier Scores, reflecting superior probabilistic accuracy and appropriate dispersion of predicted probabilities. In contrast, while Random Forest maintained good discriminative performance, it exhibited a markedly elevated calibration slope, suggesting instability in probability scaling. This distortion is a known inherent characteristic of some tree-based algorithms, which justifies the careful evaluation of calibration metrics alongside standard discrimination measures to select the most reliable model for clinical use.

**Table 3:** Integrated evaluation of discrimination, generalization, and calibration of the selected models

Model	Train AUC	Test AUC	Overfitting (Diff)	Brier score	Calibration intercept	Calibration slope
Random Forest	1.0000	0.9996	0.0004	0.0316	0.1758	3.3352
KNN	0.9999	0.9991	0.0008	0.0121	−0.7265	0.9784
LightGBM	1.0000	0.9985	0.0015	0.0129	0.4980	1.1404
SVM	0.9994	0.9984	0.0010	0.0154	0.2137	1.4598
XGBoost	1.0000	0.9971	0.0029	0.0216	0.4276	1.3509
Logistic Regression	0.9915	0.9746	0.0169	0.0638	0.1704	0.6862

*AUC*, area under the curve; *Diff*, the absolute difference between training and independent test AUC values

### Animal-level validation using leave-one-patient-out cross-validation

The model’s generalization at the biological level was evaluated using LOPO cross-validation. As presented in Table 4, while the performance metrics exhibited an expected decrease compared to the spectral-level internal test, all evaluated algorithms maintained discriminatory capability well above the level of random chance. Notably, SVM and KNN demonstrated the greatest robustness under this approach. This performance confirms that the machine learning models learned genuine, disease-specific spectral signatures in plasma, rather than overfitting to individual-specific biochemical variations.

**Table 4:** Comparative performance of the evaluated algorithms under leave-one-patient-out cross-validation

Model	Accuracy	AUC	Recall	Precision	F1-score	Kappa	MCC
Random forest	0.8833	0.7931	0.9677	0.9583	0.8679	0.9216	0.7651
XGBoost	0.8833	0.7931	0.9677	0.9583	0.8679	0.9288	0.7651
LightGBM	0.8833	0.7931	0.9677	0.9583	0.8679	0.9277	0.7651
KNN	0.8333	0.8621	0.8065	0.8065	0.8333	0.9099	0.6670
SVM	0.8833	0.8966	0.8710	0.8667	0.8814	0.9077	0.7667
Logistic regression	0.8833	0.8276	0.9355	0.9231	0.8727	0.8710	0.7656

*AUC*, area under the curve; MCC, Matthews Correlation Coefficient

### External validation

External validation was conducted to assess the models’ performance on an independent group (*n* = 15), evaluating both mean spectra and single-spectrum approaches to simulate different clinical workflows. As detailed in Table 5, the algorithms demonstrated excellent robustness. The RF, LightGBM, and XGBoost models maintained strong discriminative ability (AUC = 1.000), regardless of the spectral aggregation method used.

**Table 5:** External validation performance at the animal level: discrimination, calibration, and comparison with internal test results

Model	External AUC	External vs internal AUC (Δ)	Brier score	Calibration intercept	Calibration slope	Sensitivity	Specificity
Random forest	1.0000	0.0004	0.0269	−0.1900	1.4116	1.0000	1.0000
KNN	0.9375	−0.0616	0.1120	−0.2019	0.8413	1.0000	0.7500
LightGBM	1.0000	0.0015	0.0129	0.1067	1.0572	1.0000	1.0000
XGBoost	1.0000	0.0029	0.0280	−0.0036	1.0954	1.0000	1.0000
SVM	1.0000	0.0020	0.0616	−0.0045	0.8907	1.0000	0.8750
Logistic regression	1.0000	0.0250	0.0001	−0.0065	1.0110	1.0000	1.0000

*AUC*, area under the curve. Δ represents the difference between internal test AUC and external AUC

Although discriminative performance was comparable across models, calibration metrics revealed significant differences. LightGBM and XGBoost exhibited the most favorable calibration profiles, yielding probability estimates that better aligned with observed outcomes (showing the lowest Brier scores and calibration slopes close to ideal). These results indicate that the proposed diagnostic workflow can be effective even when using a single spectrum, and that LightGBM and XGBoost are the most reliable candidates for clinical implementation, a setting where accurate probability estimates are crucial for decision-making.

### Spectral variable importance analysis

The analysis of model-specific feature importance revealed a consistent pattern across tree-based algorithms (RF, LightGBM, and XGBoost), with dominant contributions from spectral variables located between 1042 and 1047 cm⁻^1^. In particular, wavenumbers 1045.6, 1044.1, 1047, and 1042.7 cm⁻^1^ were repeatedly identified among the top-ranked predictors. The SVM showed a similar distribution, although with slightly broader dispersion of importance weights. In contrast, KNN exhibited a distinct importance profile, with dominant contributions in the 1605–1623 cm⁻^1^ region (see Supplementary Material – Figure A1).

Projection of mean model importance back to the original spectral space confirmed the predominance of the 1040–1050 cm⁻^1^ region as the principal discriminative domain, followed by secondary contributions in the 1032–1039 cm⁻^1^ and 1746–1751 cm⁻^1^ regions. The 1040–1050 cm⁻^1^ interval is commonly associated with C–O stretching vibrations—which may relate to carbohydrates, glycoconjugates, or phosphate-containing biomolecules—whereas the 1605–1623 cm⁻^1^ region is generally attributed to Amide I vibrations, likely arising from protein secondary structures. The secondary discriminative region observed between 1746 and 1751 cm⁻^1^ corresponds to carbonyl (C=O) stretching vibrations, which may be associated with esterified lipids or phospholipid-containing compounds [40, 43].

Detailed rankings of the top 15 spectral variables for each algorithm, as well as the projected global importance values in the original spectral domain, are provided in the Supplementary Material (Additional Figures A4–A9).

**Fig. 4:**
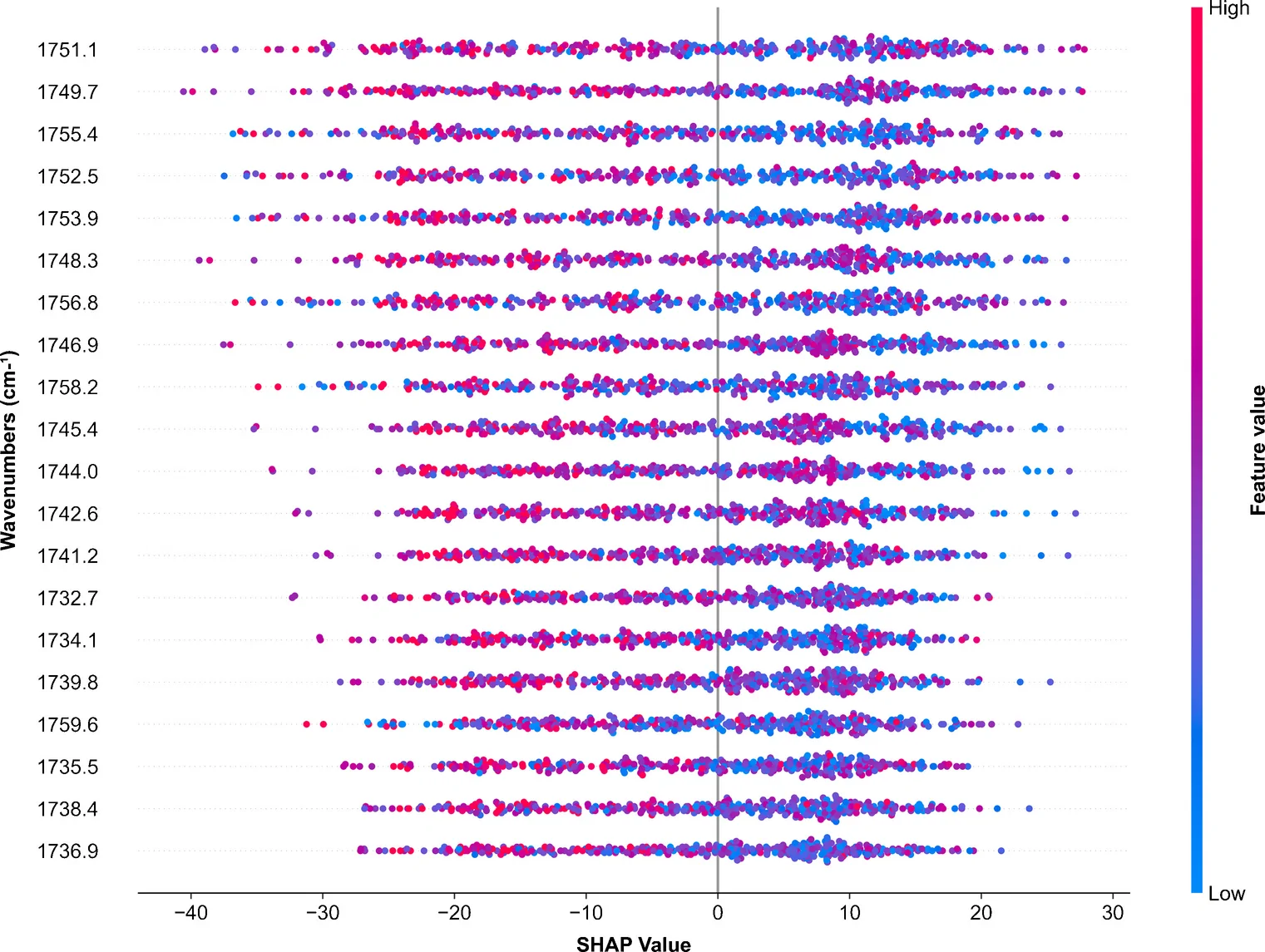
SHAP-based interpretation of the Random Forest model showing the direction and magnitude of the contribution of the most relevant spectral variables

Complementary interpretation using SHAP analysis (Fig. 4) demonstrated that the spectral variables located within the carbonyl region (1734–1751 cm⁻^1^) exerted the greatest influence on individual model predictions. Unlike global feature importance, SHapley Additive exPlanations (SHAP) additionally quantified the direction and magnitude of each variable’s contribution, showing that variations in spectral intensity within this interval consistently altered the predicted probability of disease.

## Discussion

Currently, the diagnosis of feline sporotrichosis faces an imperfect balance among speed, cost, and accuracy, particularly in endemic and resource-limited regions [6, 10, 44]. Based on a systematic review and meta-analysis of diagnostic tests for feline sporotrichosis [23], PCR demonstrates the highest pooled accuracy (AUC 0.95), but is limited by high technical complexity, operational costs, and interstudy heterogeneity. Conversely, widely used cytopathological methods show high sensitivity (~ 89%) but significantly lower specificity (~ 22%). Furthermore, traditional fungal culture creates a severe temporal bottleneck [25, 38, 45], and there are currently no validated point-of-care tests available for this neglected zoonosis [23]. However, these comparisons should be interpreted cautiously, as they rely on independent studies with distinct populations, methodological designs, and reference standards.

Addressing this critical gap, the present study demonstrates that ATR-FTIR spectroscopy applied to feline plasma, combined with supervised machine learning, provides a highly discriminatory diagnostic screening tool. The central innovation lies in its direct applicability to real-world clinical populations, eliminating the need to wait for fungal culture results or the risk of false-negative results associated with cytology. Validated both internally and through an independent external dataset, this approach supports the feasibility of a rapid, low-cost, minimally invasive, and environmentally sustainable strategy for veterinary and public health.

Although feline sporotrichosis is primarily a subcutaneous mycosis, the high fungal load and invasive nature of *Sporothrix* spp. trigger a systemic inflammatory response [46, 47]. Unlike molecular methods that target specific fungal DNA [48], infrared spectroscopy captures this global host response. The spectral “fingerprint” reflects the metabolic and immunological alterations induced by the infection, integrating changes in proteins, lipids, and carbohydrates [45, 49, 50]. Specifically, the discriminatory spectral signatures identified in this study are likely driven by the cascade of metabolic and immunological events secondary to the systemic inflammatory response [6, 13].

Because plasma is a highly heterogeneous biological matrix, these disease-induced modifications manifest as subtle changes in the relative abundance or structural organization of existing chemical constituents, rather than producing unique, isolated spectral markers [51]. Consequently, there is substantial visual overlap between the mean spectra of infected and non-infected animals. This absence of prominent global differences reinforces the essential role of supervised machine learning [45, 52]. Multivariate algorithms are required to detect and extract the localized, correlated variations distributed across specific spectral domains, enabling accurate classification where visual inspection alone fails.

Interestingly, the spectral regions identified by machine learning models as most important for disease prediction did not fully coincide with those explaining the largest proportion of overall spectral variance in the PCA. While PCA captures the dominant sources of variability in the dataset, regardless of class membership, supervised learning algorithms preferentially selected spectral domains that maximized discrimination between clinical groups [45, 53]. This distinction demonstrates that the variables accounting for the greatest variance are not necessarily those with the highest diagnostic relevance. Thus, PCA and machine learning should be viewed as complementary approaches, given that the former summarizes the global structure of the dataset, whereas the latter identifies subtle multivariate patterns associated with the disease.

Supervised algorithms model multivariate and nonlinear relationships, overcoming limitations of univariate approaches [25]. Although all models demonstrated statistically identical overall accuracy, final selection prioritized the MCC and the Brier Score. The MCC ensures balanced performance across the four categories of the confusion matrix and is less sensitive to class imbalance [54, 55]. The Brier Score evaluates probabilistic calibration, ensuring clinical reliability of predicted probabilities [56].

To improve the interpretability of the supervised learning models, SHAP analysis was performed to quantify both the magnitude and direction of the contribution of individual spectral variables to the classification process. Whereas projected feature importance consistently identified the 1040–1050 cm⁻^1^ interval as the principal discriminative region across the evaluated algorithms, SHAP analysis of the Random Forest model highlighted the carbonyl region (approximately 1734–1751 cm⁻^1^) as exerting the greatest influence on individual predictions. These approaches complement each other, providing both general and model-specific perspectives on spectral relevance, thereby enhancing the interpretability and transparency of the predictive models and strengthening the biochemical interpretation of the identified spectral signatures. Biologically, the 1040–1050 cm⁻^1^ and 1032–1039 cm⁻^1^ regions emerged as the predominant discriminative domains, possibly associated with C–O vibrations in carbohydrates and glycoproteins and with phosphate-containing structures, suggesting systemic metabolic repercussions of infection [33, 45, 57]. In the context of a systemic infection, these variations likely reflect changes in plasma glycidic components, such as the increased circulation of heavily glycosylated acute-phase proteins and immunoglobulins. Furthermore, the secondary discriminative band at 1746–1751 cm⁻^1^, attributed to C=O stretching of ester groups present in lipids and phospholipids, may indicate modifications in the systemic lipid profile driven by the host’s inflammatory response and cellular membrane remodeling [57, 58]. Collectively, these spectral signatures demonstrate that the ATR-FTIR model successfully detects the integrated biochemical and immunological host response, validating the use of plasma for diagnosing a predominantly localized disease.

To ensure the clinical reliability of the ATR-FTIR approach, we employed a tiered validation strategy. While the initial spectral-level validation demonstrated excellent analytical discrimination, it may overestimate predictive performance. Although stratified sampling preserves class proportions, it does not completely account for within-subject dependence, as repeated spectra from the same individual are not fully independent [59, 60]. To address this limitation, LOPO cross-validation provided a more conservative and biologically relevant estimate of model performance by ensuring complete separation between training and testing animals, thereby evaluating the ability of the model to generalize to previously unseen individuals [59, 61]. Finally, external validation bridged the gap to real-world application. By demonstrating that a single randomly selected spectrum per animal yielded diagnostic performance equivalent to that obtained using averaged spectra, we further supported the potential applicability of the proposed workflow in routine clinical settings, where acquiring multiple replicates is often impractical.

Both validation approaches successfully classified the independent animals, achieving complete discrimination between infected and uninfected cats. The maintenance of this performance suggests that the proposed workflow is robust and does not rely solely on the spectral mean. Despite the equivalent discriminatory performance among all evaluated algorithms, their calibration profiles differed. Notably, LightGBM and XGBoost demonstrated superior calibration, yielding probability estimates that closely aligned with observed outcomes. In diagnostic settings, such probabilistic accuracy is crucial. Well-calibrated models provide reliable estimates of the actual probability of disease, directly influencing clinical decision-making such as establishing diagnostic confidence, prioritizing confirmatory tests, or guiding the initiation of treatment [62, 63]. Therefore, calibration metrics serve as a decisive criterion for selecting the most appropriate model for clinical implementation, highlighting LightGBM and XGBoost as the most robust candidates.

From an operational standpoint, ATR-FTIR requires minimal sample preparation, small plasma volume, rapid acquisition, and no reagents or amplification kits. Once the equipment is acquired, the cost per test is low and chemical waste generation is minimal, features particularly relevant for endemic regions with limited infrastructure [36, 48].

Although these findings support the use of ATR-FTIR spectroscopy as a complementary screening tool for feline sporotrichosis, there are still limitations to be addressed. First, since the technique reflects global biochemical changes rather than pathogen-specific signatures, overlapping metabolic responses resulting from comorbidities, neoplasms, or other infectious and inflammatory diseases could interfere with spectral patterns. Second, the imperfect sensitivity of fungal culture as a reference standard may have led to false-negative results. Third, although the potential impact of repeated measurements per animal was mitigated by the use of LOPO cross-validation and animal-level external validation, and despite rigorous procedural standardization, day-to-day analytical variability across multiple sessions cannot be entirely ruled out. Finally, external validation was limited by a small dataset (*n* = 15) from a single geographic region. Therefore, future multicenter studies incorporating heterogeneous clinical populations, differential diagnoses, larger sample sizes, and batch validation strategies are essential to confirm the robustness, generalizability, and clinical applicability of the algorithms.

## Conclusion

This study demonstrates that ATR-FTIR spectroscopy applied to domestic cat plasma, combined with chemometric analysis and machine learning algorithms, exhibits promising discriminative ability and appropriate calibration for the diagnosis of feline sporotrichosis, with maintained performance in independent external validation. By identifying systemic biochemical signatures associated with infection, this approach relies on host metabolic rather than direct pathogen detection, expanding the diagnostic scope as a complementary strategy.

Considering the limitations reported in the literature for currently available methods, such as prolonged turnaround time for culture, higher cost and technical complexity for PCR, and reduced specificity for cytology, the proposed strategy emerges as a promising complementary tool for rapid screening, particularly in endemic regions with limited laboratory infrastructure. Although multicenter validation is necessary, the integration of plasma spectral fingerprinting and machine learning modeling represents a promising translational advance with the potential to strengthen early diagnosis, optimize therapeutic decision-making, and contribute to the epidemiological control of feline sporotrichosis from a One Health perspective.

## Supplementary Information

Below is the link to the electronic supplementary material.

Supplementary file1 (DOCX 2.90 MB)

## Data Availability

The datasets used and/or analyzed during the current study are available from the corresponding author on reasonable request.
