# Supplementary material for: Rapid identification of feline sporotrichosis by ATR-FTIR spectroscopy coupled with machine learning
Source: Anal Bioanal Chem. 2026 Jul 24;418(19):6485–98. doi: 10.1007/s00216-026-06671-3 (PMC13585890; doi:10.1007/s00216-026-06671-3)
Supplement: Supplementary file 1 [file 216_2026_6671_MOESM1_ESM.docx]

**Rapid Identification of Feline Sporotrichosis by ATR-FTIR Spectroscopy Coupled with Machine Learning**

**Supplementary Material**

*Figure A1* – Global spectral importance

*Figure A2* - Principal Component Loadings (PC1, PC2, and PC3) for spectral differentiation between cats with sporotrichosis and the control group.

*Figure A3* - Outlier detection using PCA: 3D projection space

*Figure A4* - Top 15 spectral variables for Random Forest

*Figure A5* - Top 15 spectral variables for KNN

*Figure A6* - Top 15 spectral variables for LightGBM

*Figure A7* - Top 15 spectral variables for SVM

*Figure A8* - Top 15 spectral variables for XGBoost

*Figure A9* - Top 15 spectral variables for Logistic Regression
